# Is West Africa Approaching a Catastrophic Phase or is the 2014 Ebola Epidemic Slowing Down? Different Models Yield Different Answers for Liberia

**DOI:** 10.1371/currents.outbreaks.b4690859d91684da963dc40e00f3da81

**Published:** 2014-11-20

**Authors:** Gerardo Chowell, Lone Simonsen, Cécile Viboud, Yang Kuang

**Affiliations:** Division of Epidemiology and Population Studies, Fogarty International Center, National Institutes of Health, Bethesda, Maryland, USA; Mathematical, Computational & Modeling Sciences Center, School of Human Evolution and Social Change, Arizona State University, Tempe, Arizona, USA; Department of Global Health, George Washington University, Milken Insittute School of Public Health, Washington DC, Maryland, USA; Division of International Epidemiology and Population Studies, Fogarty International Center, National Institutes of Health, Bethesda, Maryland, USA; School of Mathematical and Statistical Sciences, Arizona State University, Tempe, Arizona, USA

**Keywords:** ebola

## Abstract

An unprecedented epidemic of Zaire ebolavirus (EBOV) has affected West Africa since approximately December 2013, with intense transmission on-going in Guinea, Sierra Leone and Liberia and increasingly important international repercussions. Mathematical models are proving instrumental to forecast the expected number of infections and deaths and quantify the intensity of interventions required to control transmission; however, calibrating mechanistic transmission models to an on-going outbreak is a challenging task owing to limited availability of epidemiological data and rapidly changing interventions. Here we project the trajectory of the EBOV epidemic in Liberia by fitting logistic growth models to the cumulative number of cases. Our model predictions align well with the latest epidemiological reports available as of October 23, and indicates that the exponential growth phase is over in Liberia, with an expected final attack rate of ~0.1-0.12%. Our results indicate that simple phenomenological models can provide complementary insights into the dynamics of an outbreak and capture early signs of changes in population behavior and interventions. In particular, our results underscore the need to treat the effective size of the susceptible population as a dynamic variable rather than a fixed quantity, due to reactive changes in transmission throughout the outbreak. We show that predictions from the logistic model are more variable in the earlier stages of an epidemic (such as the EBOV epidemics in Sierra Leone and Guinea). More research is warranted to compare the performances of mechanistic and phenomenological approaches for disease forecasts, before such predictions can be fully used by public health authorities.

## Commentary

An unprecedented epidemic of Zaire ebolavirus (EBOV) has affected West Africa since approximately December 2013, with intense transmission on-going in Guinea, Sierra Leone and Liberia and increasingly important international repercussions 1. As in previous international health crises such as the SARS epidemic and the 2009 influenza pandemic, mathematical models are proving instrumental to forecast the expected number of infections and deaths, quantify the intensity of interventions needed to achieve control (e.g., Ebola treatment centers, protective kits) and monitor the benefits of these interventions once they have been initiated. Calibrating mechanistic transmission models to the current Ebola outbreak is a challenging task however, complicated by underreporting of infections and deaths, population heterogeneity in infection risk 2, changes in population behavior and intervention strategies that blunt the transmission rate of the virus after the initial phase of the epidemic, and stochastic effects 3.

Here we build on a recent modeling study published by Lewnard et al. to contrast predictions derived from traditional mechanistic models and phenomenological approaches 4. Lewnard et al calibrated a mechanistic model of EBOV transmission to epidemiological reports of cases and deaths in the greater Monrovia area, the capital city of Liberia, during the early phase of the epidemic, June 14 to September 23, 2014. This study has produced by far the most dire outlook yet on the Ebola outbreak, suggesting that if nothing is done above and beyond current interventions, about 27% of the Monrovia population will be infected with EBOV before the epidemic runs its course towards the end of 2015. Lewnard et al’s model is based on six estimated parameters, including the basic reproduction number Ro, estimated to be particularly high (R0=2.4). The model showed signs of overestimating the trajectory of the epidemic by mid-October 2014 (Figure 1 in 4). In parallel, the Liberia national response team has reported that the EBOV outbreak has slowed considerably in recent weeks 10, before the heightened international response kicked in, probably as a result of social distancing measures, patient isolation and changes in funeral practices 5. Public health experts have confirmed the epidemic slowdown in at least two counties of Liberia in a series of recent reports^10^^,^ 14 ^,^ 15.

When few epidemiological data are available to fit mechanistic models, and health behavior and interventions are rapidly changing, standard model theory can quickly overshoot the trajectory of an epidemic. In this commentary, we propose a complementary method to project the temporal course of an epidemic, which is based on fitting a logistic growth curve to time series of cumulative case counts^7 ^ -- a phenomenological approach that is less data hungry and does not make complex mechanistic assumptions about the transmission process. We illustrate this approach with data from the rapidly changing epidemic in Liberia, and provide additional estimates for Sierra Leone and Guinea.

The logistic growth model provides a statistical description of the outbreak trajectory, inspired from population biology^7^ . This model has two fitted parameters and follows the equation C’(t) = rC(1-C/K) where C’(t) is the rate of change in the cumulative number of total Ebola cases comprising suspected, probable and confirmed cases, “r" is the intrinsic growth rate ( per unit of time) and K is the final epidemic size (total no. of cases) 7 ^,^ 11.The logistic growth model assumes a saturation effect, so that as more EBOV cases accumulate, the size of the at-risk susceptible population decreases, implicitly capturing behavior changes (e.g., campaigns to educate the population on how to avoid contracting the disease), initiation of public health interventions, and increasing use of diagnostic tests to quickly identify and isolate EBOV cases 6 ^,^ 13. As a case in point of a rapidly changing outbreak landscape, empirical estimates of the reproduction number decreased from 12 during the first generation of the EBOV outbreak in Nigeria to less than one during the second and third generations due to rapid initiation of public health measures 16.

The logistic growth model fitted to the early phase of the Ebola epidemic in Liberia yields modest predictions of the final epidemic size of around 5,000 (95%CI: 4,574; 5,445) reported cases, corresponding to population attack rates of ~0.10%-0.12% (Figure 1, Liberia population size=4.4 millions). Specifically, the model is fitted by least-squares to 26 daily observations of cumulative case counts, starting from June-18, 2014 (red dots) and provides a good approximation of the course of the epidemic thus far in Liberia, including the 14 most recent daily case reports (blue dots in Figure 1). These predictions provide a more optimistic outlook on the epidemic in Liberia, which aligns better with the latest WHO epidemiological reports available as of October 23, 2014 (9 and Figure 1).

Although the logistic growth model is phenomenologic, it can provide implicit information about the transmission process as estimates of the reproduction number can be derived. The basic reproduction number Ro is given by Ro = exp(r*T), where r is the intrinsic growth rate (estimated as before) and T is the mean generation interval. Further, the effective reproduction number at time t is given by R(t)= (1-(C(t)/K)))*exp(r*T), where K is the mean final epidemic size (estimated as before). Logistic growth model estimates for the EBOV outbreak in Liberia support a decrease in transmission around September 6, 2014, with the reproduction number declining from Ro=2.4 at the beginning of the epidemic in August, to R=1.7 around September 6, to R=1.3 by October 1, assuming a generation interval of 15 days 8 ^,^ 12.

To check the validity of the logistic model predictions in different settings, we fitted the model to data from Sierra Leone and Guinea, tow countries with active Ebola transmission. Figures 2 and 3 show that final size predictions are consistently lower than those of a simple SEIR-type model in exponential growth mode, as the logistic curve captures a slowdown in transmission in both countries. We note however that final size predictions are more variable for Sierra Leone and Guinea as estimates keep increasing with inclusion of additional observations, suggesting that EBOV transmission is still high in these countries and the outbreak is far from over. Our estimates for the effective reproduction number on October 1, 2014 are 1.2 in Sierra Leone and 1.4 in Guinea. We also note that the logistic curve is unable to capture the biphasic nature of the outbreak in Guinea, resulting from the asynchronous dissemination of EBOV in different areas of the country. Hence here we only fit the most recent phase of the epidemic in Guinea (Figure 2).

**Liberia:**
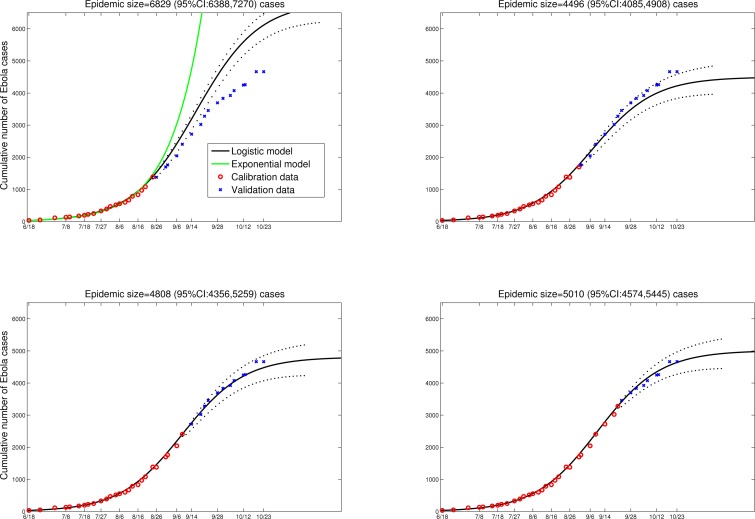
Predictions of the cumulative number of Ebola cases in Liberia by the logistic growth equation C’(t) = rC(1-C/K) where C’(t) is the rate of change in the cumulative number of total Ebola cases comprising suspected, probable and confirmed cases, “r” is the intrinsic growth rate (1/day) and K is the final epidemic size. The total number of Ebola cases satisfies essentially a density dependent exponential equation, with an exponent linearly decreasing as a function of the total cases reported. The two parameters “r” and “K” were estimated from the early epidemic phase by numerical optimization. Model fits (A-D) are shown for an increasing number of data points used for model calibration. The black solid lines correspond to C(t), the predicted cumulative number of Ebola cases. Blue circles are future cases used for model validation. The exponential model fit (green solid line) is shown as a reference for a worst-case scenario in panel A. We model here the national epidemic curve for Liberia, which follows a similar epidemic pattern to that of Montserrado county, the subset analyzed by Lewnard et al. The dotted lines correspond to the 95% confidence intervals of the prediction curve provided by the model.

**Guinea:**
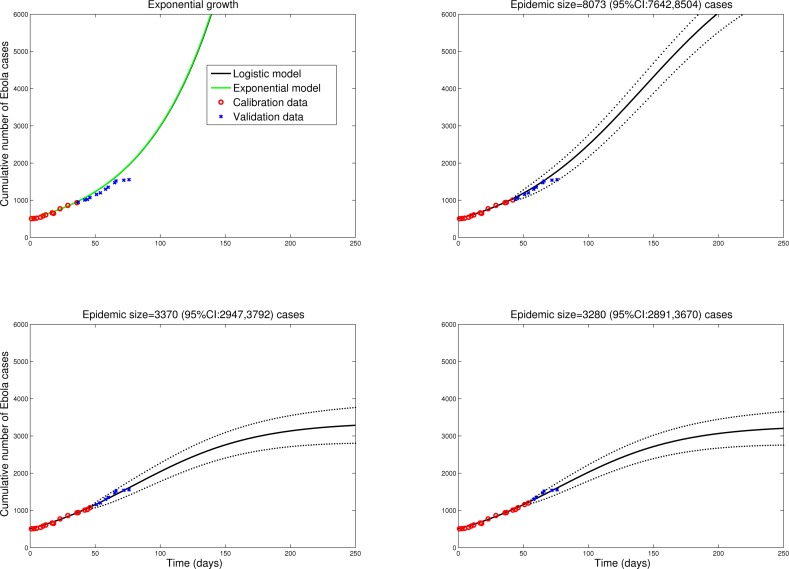
Predictions of the cumulative number of Ebola cases in Guinea by the logistic growth equation (see Figure 1 or text for equation). Model fits (A-D) are shown for an increasing number of data points used for model calibration. The black solid lines correspond to C(t), the predicted cumulative number of Ebola cases. Blue circles are future cases used for model validation. We model here the second phase of the national epidemic curve for Guinea (Guinea has a biphasic epidemic due to spatial heterogeneity in EBOV transmission).

**Sierra Leone:**
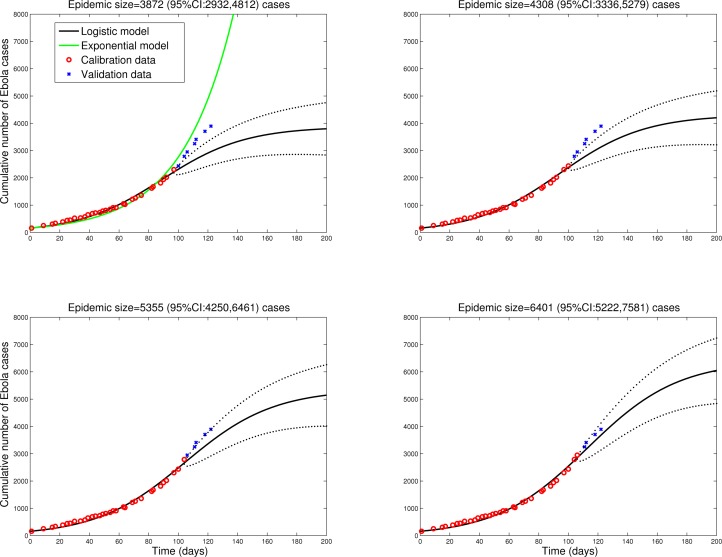
Predictions of the cumulative number of Ebola cases in Sierra Leone by the logistic growth equation (see text or Figure 1 for equation). Model fits (A-D) are shown for an increasing number of data points used for model calibration. The black solid lines correspond to C(t), the predicted cumulative number of Ebola cases. Blue circles are future cases used for model validation. The dotted lines correspond to the 95% confidence intervals of the prediction curve provided by the model.

**Conclusion**

Predicting the final epidemic size of an ongoing epidemic is never an easy task. Here we show that simple phenomenological models can be used along traditional mechanistic transmission models to check the validity of predictions when observations are few, and capture rapid changes in transmission intensity during an epidemic. Our results suggest the need to model the total effective susceptible population size as a dynamic variable instead of a fixed quantity, which may be particularly important for the on-going EBOV outbreak. In the case of the EBOV epidemic in Liberia, our forecasting results based on the logistic growth model support a decline in the effective size of the at-risk susceptible population, a process likely dominated by changes in population behavior and the impact of public health interventions. In contrast, the pessimistic predictions by Lewnard et al. 4 assumed that the epidemic was still in an exponentially growing phase as of October 15, 2014. The more complex mechanistic approach used by Lewnard. 4 et al may have missed a recent decline in transmission intensity due to the large number of parameters fitted with respect to the amount of data (6 parameters, 10 observations).

While recent field reports and logistic growth model forecasts support that Ebola transmission is currently slowing down in Liberia^10^ , we cannot rule out case reporting bias, future waves of infection as interventions and population behavior are relaxed, or a switch to endemicity. Indeed, the logistic model was unable to accurately capture the bimodal nature of the outbreak in Guinea, and forecasts were less stable for the earlier stages of an epidemic, as in Sierra Leone.

Mechanistic transmission models are ultimately preferable to phenomenological approaches for a variety of reasons. Most importantly, they provide an explicit description of the transmission process, and hence can be used to test the effect of interventions. However, when observations are too few to calibrate complex models, it may be useful to consider alternative approaches in parallel to contrast and compare early predictions. Further, we have shown here that a phenomenological approach such as the logistic growth model can provide insights into the transmission process (for instance, by capturing the effective reproduction number R(t)). We hope that this early work will stimulate further research into the upsides and downsides of mechanistic and phenomenological approaches in a variety of outbreak settings, and on the bridges between these approaches (as we have shown here for reproduction number estimates). It is unfortunate that infectious disease forecasting crucially lags behind other scientific domains such as weather predictions (but see interesting advances in 17).

Our work is preliminary and prone to a number of limitations. As in previous forecasting studies 17, our approach makes the assumption of homogeneously mixed epidemics in Liberia, Guinea and Sierra Leone, which is unrealistic (especially in Guinea, where EBOV transmission is asynchronous in different parts of the country). Future work should focus on testing different forecasting approaches on real-time district-level data, which are currently lacking in the public domain. Finally, as in previous work 4 ^,^ 17, we have used cumulative case time series rather than daily incidences, which is required to smooth uncertainties in the reporting process and stochastic noise.

There is still considerable uncertainty about the future of the Ebola epidemic in West Africa and a large amount of work is warranted to improve forecasting tools to a point where they can be routinely used by public health authorities. Importantly, even if our optimistic predictions are correct for Liberia, there are compelling reasons for maintaining existing measures as the vast majority of the population remains immunologically susceptible. The implementation of additional interventions in Liberia, such as US donation of 1,700 hospital beds and influx of international health care workers can only maximize the benefits of existing interventions, relieve an overly strained health infrastructure, and help with the backlog of competing health priorities that have been sidelined by the Ebola outbreak. Further, the mop-up period for EBOV could be long given the geographical extent of the current outbreak, and it could be months before the risk of reintroduction from neighboring countries declines to zero. Hence it is likely that the international aid will be crucially needed in Liberia and in the broader region for months to come.

## Competing Interests

The authors have declared that no competing interests exist
